# Addressing child under nutrition: can traditional practices offer a solution?

**DOI:** 10.1080/16549716.2017.1327255

**Published:** 2017-06-07

**Authors:** Sarika Chaturvedi, Joanna Raven, Bhushan Patwardhan

**Affiliations:** ^a^ Centre for Complementary and Integrative Health, Interdisciplinary School of Health Sciences, Savitribai Phule Pune University, Pune, India; ^b^ Department of International Public Health, Liverpool School of Tropical Medicine, Liverpool, UK

**Keywords:** Child care, nutrition, public health, traditional medicine, massage, complementary therapy, infant care, child nutrition

## Abstract

Child under nutrition continues to be a challenge to global development, especially in resource-poor contexts. In the multifaceted attempts to address this challenge, popular traditional practices, although closely linked to infant health, remain neglected and less researched. The World Health Organization’s recent strategy on traditional medicine systems provides overarching guidance in this regard. In this commentary, we attempt to exemplify this. We highlight the importance of traditional child care practices with regard to child nutrition and the need for trans-disciplinary research to explore the potential of these for public health. Infant oil massage appears to be a potentially beneficial practice for child nutrition. Rigorous trans-disciplinary research on traditional infant massage can provide simple solutions to address child under nutrition and nurture human capabilities globally.

## Background

Global progress in improving child health indicators since 1990 has been promising, yet each year six million children worldwide die before the age of five [1]. The global target for Sustainable Development Goal 3 (SDG 3) is to reduce under-five mortality to less than 25 per 1000 births [1]. Most child deaths (80%) occur in Sub-Saharan Africa and Asia and under nutrition is the underlying cause for half of these deaths. Nearly two thirds of undernourished children in the world live in Asia and Africa, underscoring the importance of reducing under nutrition in these parts of the world for achievement of SDG 3. Several programmes and interventions aimed at nutrition and health such as breast feeding, supplementary feeding, micronutrient fortification, therapeutic feeding, and prevention and management of infectious diseases have been implemented in these countries for many years, although much remains to be achieved. The conceptual frameworks for understanding and addressing child nutrition portray its multifactorial nature [2]. However, interventions and programmes often seem to be fragmented across sectors and age groups. There is more focus on interventions targeted at improving nutrition through provision of food supplements and complementary feeds while considerably less emphasis has been placed on cultural factors that are strongly linked to child-rearing practices. Proponents of sustainable development urge traditions and cultural practices to be viewed as integral to overall social and economic development, rather than detrimental to it [3]. Countries with strong cultural heritage have the option to choose indigenous solutions to their problems [4]. In Asia and Africa, where malnutrition prevails, societies continue to follow more traditional child-rearing practices that are likely to positively affect child health outcomes. Many traditional practices are rooted in the traditional medicine of the region. The role of traditional medicine in addressing population health needs is gaining recognition as reflected in the recent World Health Organization (WHO) strategy on traditional and complementary medicine [5]. The strategy aspires to help healthcare leaders develop solutions that contribute to a broader vision of health. One of the aims of the strategy is to promote universal health coverage by integrating traditional and complementary services and self-healthcare into national health systems. We believe that substantiating the strategic guidance with relevant ideas for promotion of traditional medicine to address national health challenges would be useful to trigger envisioned actions. In this article we discuss the potential of traditional medicine to address public health challenges, using the example of oil massage of infants to address under nutrition in India.

## Current situation of child nutrition in India

India has a large young population and is also home to one third of the world’s undernourished children with over a third of under-five children being either stunted or wasted (38%) [6]. Given that undernourished children are more susceptible to infections and face a higher risk of death, it is not surprising that India has an unacceptably high infant mortality. The under-five mortality rate in India stands at 47.7 per 1000 births amounting to 1.2 million child deaths each year [7], and nearly half of these deaths are attributable to under nutrition. Besides increased risk of mortality, under nutrition in early life has irreversible effects and serious health consequences such as impaired cognitive function which can result in national economic and productivity loss. Addressing the challenge of under nutrition in India is therefore imperative for sustainable development nationally and globally. Although India has made some progress in reducing under nutrition – stunting among children under 5 years of age decreased from 48% in 2005/06 to 39% in 2015 [6] – India remains off course for achieving the World Health Assembly targets for child nutrition. This situation points to potential problems with current strategy, interventions and their implementation, and the need for new measures for improved effectiveness. Many of the current interventions are based on evidence drawn from modern medical science; however, anthropological evidence and that from traditional medicine seem to be scarce and rarely considered. Currently health education messages from the formal health system emphasize good practices such as early initiation of breast feeding and exclusive breast feeding for six months, and discourage certain practices including early weaning. However, the popular traditional practice of oil massage to the newborn and infant is not currently recommended.

## Infant oil massage

Infant massage is widely practised in Indian and other Asian settings and is commonly performed by mothers or other family members using oil. Research shows topically applied oil can be absorbed in neonates and is available for nutritional purposes [8]. Effects of massage with or without oil have been documented. Application of coconut oil to very low birth weight infants is known to augment the mechanical barrier function of the skin. It reduces transepidermal water loss (TEWL) (g/m(2)/h, Mean Difference (MD) −6.80; 95% Confidence Interval (CI) −3.48, −10.15) with better skin condition and lower bacterial growth in the oil group (20% vs. 60%) and it prevents hypothermia [9]. Other positive effects include: weight gain through stimulation of the branches of the vagus nerve resulting in increased gastric activity and insulin secretion [10]; improved brain development and reduced risk of retinopathy of prematurity [11]; and conservation of heat and energy for growth [12]. Evidence from the developing world including studies from Bangladesh, Egypt, India, and Pakistan shows emollient therapy to be associated with improved weight gain and reduced risk of infection and mortality in preterm neonates [13]. A recent review indicates a favourable effect of massage on infant health outcomes, including physical health such as weight (MD −965.25 g; 95% CI −1360.52 to −569.98) and length (MD −1.30 cm; 95% CI −1.60 to −1.00), 24-hour sleep duration (MD −0.91 hr; 95% CI −1.51 to −0.30), time spent crying (MD −0.36; 95% CI −0.52 to −0.19), decreased levels of blood bilirubin (MD −38.11 mmol/L; 95% CI −50.61 to −25.61), and fewer diarrhoea cases (Risk Ratio 0.39; 95% CI 0.20 to 0.76). The review also reported beneficial effects of massage on mental health/development such as fine/gross motor skills (Standardized Mean Differences (SMD) −0.61; 95% CI −0.87 to −0.35/SMD −0.44; 95% CI −0.70 to −0.18), social behavior (SMD −0.90; 95% CI −1.61 to −0.18), and psychomotor development (SMD −0.35; 95% CI −0.54 to −0.15) [14]. The review indicated the need for more studies and improved trials, especially in community settings in resource-poor countries and amongst term infants.

The traditional practice of oil massage in India has its roots in the ancient traditional medicine of Ayurveda. The Ayurvedic literature recommends oil massage to infants until one year to promote healthy development [15]. Massage using sesame oil-based medicated oils is recommended by Ayurveda practitioners to prevent and treat several ailments including under nutrition. However, how this affects child nutrition and growth is not known. The scarcity of evidence on traditionally practised and Ayurveda-based infant oil massage points to the need to systematically study the potential of this practice for healthy growth and development. With sufficient scientific evidence, the practice of oil massage to infants can be promoted as a public health intervention. This intervention is simple, low-cost, already acceptable to families and communities, and can therefore be scaled up easily. Learning from traditional wisdom through co-generation of knowledge by experts in both traditional medicine and biomedicine is important for realizing this potential for public health.

## Conclusion

High-quality trans-disciplinary research with an integrative approach to health can be a simple yet promising way to address present challenges of under nutrition and under-five mortality. With its advantages of acceptability, simplicity, and affordability, infant oil massage could emerge as an effective traditional medicine solution to complement efforts to address child under nutrition globally and thus also accelerate progress towards achieving SDG 3.

## Paper context

The potential of traditional practices in addressing public health challenges is gaining recognition. Infant oil massage, a popular traditional practice in the global South, appears to be a beneficial intervention to address child under nutrition. High-quality trans-disciplinary research to study the putative benefits of infant oil massage is needed for possible translation to public health.
